# Supplementary material for: Comparative genomics provides new insights into the diversity, physiology, and sexuality of the only industrially exploited tremellomycete: Phaffia rhodozyma
Source: BMC Genomics. 2016 Nov 9;17:901. doi: 10.1186/s12864-016-3244-7 (PMC5103461; doi:10.1186/s12864-016-3244-7)
Supplement: Additional file 6: — List of orphan genes with links to PFAM (related to Additional file 1: Table S1). (ZIP 1428 kb) [file 12864_2016_3244_MOESM6_ESM.zip › BLAST_HTML_FTR/G00597_P.html]

BLAST Search Results


```
BLASTP 2.2.27+


Reference:
Stephen F. Altschul, Thomas L. Madden, Alejandro A. Schäffer,
Jinghui Zhang, Zheng Zhang, Webb Miller, and David J. Lipman (1997),
"Gapped BLAST and PSI-BLAST: a new generation of protein database
search programs", Nucleic Acids Res. 25:3389-3402.


Reference for
composition-based statistics:
Alejandro A. Schäffer, L. Aravind, Thomas L. Madden, Sergei
Shavirin, John L. Spouge, Yuri I. Wolf, Eugene V. Koonin, and
Stephen F. Altschul (2001), "Improving the accuracy of PSI-BLAST
protein database searches with composition-based statistics and
other refinements", Nucleic Acids Res. 29:2994-3005.


Database: nr
           71,551,133 sequences; 26,053,659,533 total letters


Query= G00597_P

Length=297
                                                                      Score     E
Sequences producing significant alignments:                          (Bits)  Value

emb|CED82576.1|  hypothetical protein [Xanthophyllomyces dendrorh...   600    0.0  
emb|CEG82267.1|  hypothetical protein RMATCC62417_16365 [Rhizopus...  43.5    0.19 


 >emb|CED82576.1| hypothetical protein [Xanthophyllomyces dendrorhous]
Length=296

 Score =  600 bits (1547),  Expect = 0.0, Method: Compositional matrix adjust.
 Identities = 296/296 (100%), Positives = 296/296 (100%), Gaps = 0/296 (0%)

Query  1    MAAFASTLDRTEKTTEDVGLESALTEEPSRYSNPLATPGKDGDHSGSCRPSLTFANQTGR  60
            MAAFASTLDRTEKTTEDVGLESALTEEPSRYSNPLATPGKDGDHSGSCRPSLTFANQTGR
Sbjct  1    MAAFASTLDRTEKTTEDVGLESALTEEPSRYSNPLATPGKDGDHSGSCRPSLTFANQTGR  60

Query  61   RVSYTQISVLDNVPEEDDSDGSHIFDGLGEAVESGSGPKSAMLGYRAWRRDSAASATDAT  120
            RVSYTQISVLDNVPEEDDSDGSHIFDGLGEAVESGSGPKSAMLGYRAWRRDSAASATDAT
Sbjct  61   RVSYTQISVLDNVPEEDDSDGSHIFDGLGEAVESGSGPKSAMLGYRAWRRDSAASATDAT  120

Query  121  VDELEPCPISAFNQVSPLHDSFQPSFGMFDDFRFSSLDSIPTNLPLSLSSISPARPGTAE  180
            VDELEPCPISAFNQVSPLHDSFQPSFGMFDDFRFSSLDSIPTNLPLSLSSISPARPGTAE
Sbjct  121  VDELEPCPISAFNQVSPLHDSFQPSFGMFDDFRFSSLDSIPTNLPLSLSSISPARPGTAE  180

Query  181  SSFSQDESFETPNTPGWSPRHSGSYAGPPPTGVSSRSTRSSAPVIHISNQLESSRASSFD  240
            SSFSQDESFETPNTPGWSPRHSGSYAGPPPTGVSSRSTRSSAPVIHISNQLESSRASSFD
Sbjct  181  SSFSQDESFETPNTPGWSPRHSGSYAGPPPTGVSSRSTRSSAPVIHISNQLESSRASSFD  240

Query  241  PVQKLMEYQINTFAGKDLLESPWVDETSPSMLVDTSLSKLEQKMSAADKEGSNQKL  296
            PVQKLMEYQINTFAGKDLLESPWVDETSPSMLVDTSLSKLEQKMSAADKEGSNQKL
Sbjct  241  PVQKLMEYQINTFAGKDLLESPWVDETSPSMLVDTSLSKLEQKMSAADKEGSNQKL  296


>emb|CEG82267.1| hypothetical protein RMATCC62417_16365 [Rhizopus microsporus]
Length=508

 Score = 43.5 bits (101),  Expect = 0.19, Method: Compositional matrix adjust.
 Identities = 33/113 (29%), Positives = 51/113 (45%), Gaps = 6/113 (5%)

Query  187  ESFETPNTPGWSPRHSG----SYAGPPPTGVSSRSTRSSAPVIHISNQLESSRASSFDPV  242
            ESF TP T G S  +      SY  P P  V S        +I+    L + R       
Sbjct  12   ESFCTPETLGTSVNYVNLLLTSYGYPVPIVVDSSDPEDKCKIINCIYALINDRKKDMQEK  71

Query  243  QKLMEYQINTF-AGKDLLESPWVDETSPSMLVDTSLSKLEQKMSAADKEGSNQ  294
            Q+L+E +IN     ++ L++   +     ML D  +S+L+ K+ A++KE   Q
Sbjct  72   QQLLE-RINQLDKNQEELQTLLANHKREYMLQDKRISELKTKLDASEKEQKRQ  123


Lambda      K        H        a         alpha
   0.310    0.126    0.364    0.792     4.96 

Gapped
Lambda      K        H        a         alpha    sigma
   0.267   0.0410    0.140     1.90     42.6     43.6 

Effective search space used: 2278096025968


  Database: nr
    Posted date:  Sep 23, 2015 12:05 AM
  Number of letters in database: 26,053,659,533
  Number of sequences in database:  71,551,133


Matrix: BLOSUM62
Gap Penalties: Existence: 11, Extension: 1
Neighboring words threshold: 11
Window for multiple hits: 40
```
